# OUTCOMES OF PATIENTS UNDERGOING PANCREATODUODENECTOMY (WHIPPLE’S PROCEDURE) ACCORDING TO THE PRESENCE OF PREOPERATIVE BILIARY DRAINAGE

**DOI:** 10.1590/S0004-2803.24612024-089

**Published:** 2025-07-21

**Authors:** Rómulo VARGAS-RUBIO, Ana Maria LEGUIZAMO-NARANJO, Oscar MUÑOZ-VELANDIA, Cristiam David PULGARIN-HERRERA, Carlos Ernesto LOMBO, Liliana CUEVAS-LOPEZ, Elio Fabio SANCHEZ, Francisco Javier HENAO

**Affiliations:** 1 Gastroenterology Unit, Hospital Universitario San Ignacio, Bogotá D.C., Colombia.; 2 Department of Internal Medicine, Pontificia Universidad Javeriana, Bogotá D.C., Colombia.; 3 Department of Internal Medicine, Hospital Universitario San Ignacio, Bogotá D.C., Colombia.; 4 Gastroenterology Fellowship Program, Pontificia Universidad Javeriana, Bogotá D.C., Colombia.; 5 Department of Surgery, Hospital Universitario San Ignacio, Pontificia Universidad Javeriana, Bogotá D.C., Colombia.

**Keywords:** Drainage, adverse effects, choangiopancreatography, endoscopic retrograde, pancreaticoduodenectomy, jaundice, obstructive, etiology, pancreatic neoplasms, common bile duct neoplasms, preoperative care, Drenagem, efeitos adversos, colangiopancreatografia, retrograda endoscópica, pancreatoduodenectomia, icterícia, obstrutiva, etiologia, neoplasias pancreáticas, neoplasias do ducto biliar comum, cuidados pré-operatórios

## Abstract

**Background:**

Pancreaticoduodenectomy is the procedure of choice for the treatment of resectable pancreaticoduodenal tumours. It has been proposed that jaundice is associated with worse outcomes, but the usefulness of preoperative biliary drainage in these patients is still controversial.

**Methods:**

Retrospective cohort study of patients undergoing Whipple procedure at the Hospital Universitario San Ignacio, Bogotá (Colombia), between January 2010 and June 2023. The cohort of patients who underwent preoperative biliary drainage was compared with those who went directly operated on. Comorbidities, functional status and procedural characteristics were recorded. The outcomes, including mortality and intraoperative and 30-day postoperative complications, were compared between groups.

**Results:**

A total of 98 patients were included, 49 of whom underwent preoperative biliary drainage. In this group, there was a higher proportion of patients with pathological stage II and III disease (77.5 vs 49.0, *P*=0.04) and higher preoperative bilirubin levels (median 6.4 vs 4.9 mg/dL; *P*=0.02). There were no differences in intraoperative (10.2% vs 14.3%; *P*=0.34) or postoperative (61.2% vs 51%; *P*=0.15) complications, but 30-day mortality was higher in patients with biliary drainage (8.2 vs 20.4%; *P*=0.03).

**Conclusion:**

Our data suggest that there are no differences in postoperative complications. The higher mortality rate in patients with preoperative biliary drainage may be related to differences in baseline patient characteristics and/or delays between biliary drainage and Whipple procedure.

## HIGHLIGHTS

• The usefulness of preoperative biliary drainage in patients undergoing Whipple procedure is still controversial.

• Our data suggest that there are no differences in postoperative complications.

• 30-day mortality is higher in patients with biliary drainage maybe related to differences in baseline characteristics and/or delays between biliary drainage and Whipple procedure.

## RESUMO

**Contexto:**

A pancreatoduodenectomia é o procedimento de escolha para o tratamento de tumores pancreato-duodenais ressecáveis. Foi proposto que a icterícia está associada a piores desfechos, mas a utilidade da drenagem biliar pré-operatória nesses pacientes ainda é controversa.

**Métodos:**

Estudo de coorte retrospectivo de pacientes submetidos ao procedimento de Whipple no Hospital Universitário San Ignacio, Bogotá (Colômbia), entre janeiro de 2010 e junho de 2023. A coorte de pacientes que se submeteram à drenagem biliar pré-operatória foi comparada com aqueles que foram operados diretamente. Comorbidades, estado funcional e características do procedimento foram registrados. Os desfechos, incluindo mortalidade e complicações intraoperatórias e pós-operatórias em 30 dias, foram comparados entre os grupos.

**Resultados:**

Um total de 98 pacientes foi incluído, dos quais 49 se submeteram à drenagem biliar pré-operatória. Neste grupo, houve uma maior proporção de pacientes com doença em estágio patológico II e III (77,5 vs 49,0, *P*=0,04) e níveis de bilirrubina pré-operatória mais altos (mediana 6,4 vs 4,9 mg/dL; *P*=0,02). Não houve diferenças nas complicações intraoperatórias (10,2% vs 14,3%; *P*=0,34) ou pós-operatórias (61,2% vs 51%; *P*=0,15), mas a mortalidade em 30 dias foi maior em pacientes com drenagem biliar (8,2 vs 20,4%; *P*=0,03).

**Conclusão:**

Nossos dados sugerem que não há diferenças nas complicações pós-operatórias. A maior taxa de mortalidade em pacientes com drenagem biliar pré-operatória pode estar relacionada a diferenças nas características basais dos pacientes e/ou atrasos entre a drenagem biliar e o procedimento de Whipple.

## INTRODUCTION

Whipple procedure, also known as pancreatoduodenectomy, is currently the surgical procedure of choice for the resection of pancreatic and periampullary tumors^1^, being also useful in other scenarios such as chronic pancreatitis with inflammatory mass formation in the pancreatic head^2^, pancreatitis due to trauma^2^, neuroendocrine tumors, metastases, cholangiocarcinoma, mesenchymal tumors, duodenal adenocarcinomas and serous cystadenomas^3^. The standard procedure consists of resection of the duodenum, distal common bile duct, gallbladder, pancreatic head and antrectomy^4^ ^-^ ^6^.

Despite its widespread use, it is a procedure with high morbidity, and in high volume centers, complications and mortality rates may reach values close to 50% and <5%, respectively^5^. One of the factors negatively associated with surgical outcome is obstructive jaundice at the time of surgery, as it has been described that prolonged cholestasis induces a procoagulant state, decreasing plasminogen activating factor-dependent fibrinolytic activity and causing a disruption of the mucosal barrier, increasing endotoxin absorption and the production of proinflammatory interleukins^7^ ^,^ ^8^.

Following the biological plausibility of the detrimental effects of jaundice, Dr Allen Whipple introduced the concept and practice of preoperative biliary drainage in 1935, which evolved with the advent of transparihepatic drainage and endoscopic retrograde cholangiopancreatography (ERCP) with placement of biliary prostheses^9^. Although there are studies that support the routine use of preoperative biliary drainage^10^ ^-^ ^12^, it is still controversial whether this premise is true, as some studies have yielded contradictory results, even showing higher rates of complications compared to patients taken to early surgery^13^. One of the studies that has had the greatest impact on this controversy is the DROP trial, which found higher rates of complications in the preoperative biliary drainage groups (74% vs 39%), as well as higher rates of operative complications (47% vs 37%), with no difference in mortality^8^.

Considering that preoperative biliary drainage is still a subject of debate, the objective of this study is to describe the clinical and demographic aspects of patients undergoing pancreaticoduodenectomy and to evaluate the outcomes according to the presence of preoperative biliary drainage, based on the experience of a reference hospital in Colombia.

## METHODS

An analytical observational study based on a retrospective cohort was carried out, including all patients over 18 years of age who underwent pancreatoduodenectomy at the Hospital Universitario San Ignacio, Bogota, Colombia, between January 2010 and June 2023. Exclusion criteria were: patients referred to another hospital postoperatively and patients with chemotherapy administration in the month prior to surgery. This protocol was endorsed by the institutional research committee (FM-CIE-1030-23).

All patients undergoing pancreatoduodenectomy in the study period were identified from an institutional registry in which all procedures performed by the gastrointestinal surgery unit are systematically recorded. Sociodemographic data were systematically collected during patient care. Variables related to comorbidities, laboratory results, patient functionality, periprocedural and procedural characteristics, intraoperative and postoperative complications were systematically recorded using a standardized format. Information on the requirement for unplanned reintervention and 30-day readmission, hospital stay and 30-day death was collected from the information recorded in the institutional electronic medical records.

Cohorts were divided according to the performance or not of preoperative biliary drainage. Preoperatory biliary drainage was defined as biliary drainage by ERCP plus biliary stenting or percutaneous drainage by interventional radiology. 

The Charlson Index^14^, Karnosky index^15^, functional status according to the Eastern Cooperative Oncology Group (ECOG)^16^, and the American Society of Anesthesiologists (ASA) physical status classification ^17^ were evaluated. The Subjective Global Assessment (SGA) was used to categorize the nutritional status into three groups, moderately malnourished (A), suspected malnutrition (B), or severe malnutrition (C)^18^.

Intraoperative complications were defined as bleeding ≥1000 cc during surgery and vascular or hollow viscera lesions during the procedure. For postoperative complications definition, the Claven-Dindo classification was used^19^. It included surgical site infections (SSI) categorized as superficial (subcutaneous cellular tissue), deep (fascia/muscle) and organ/space^20^; biliary leak confirmed by ERCP or intraoperative cholangiography; pancreatic fistulas categorized as Grade A (Transient fistula with no clinical or management impact), Grade B (Requirement for endoscopic or percutaneous drainage/need for angiographic procedures due to fistula-related bleeding), Grade C (Associated organ failure)^21^; multi-organ failure, need for mechanical ventilation postoperatively, need for ICU stay and unplanned reoperation, readmission and mortality during the first 30 days postoperatively.

### Statistical analysis

Absolute and relative frequencies were reported to describe qualitative variables. Measures of central tendency and dispersion were calculated for quantitative variables. Mean and standard deviation were used for variables with normal distribution, and median and interquartile range for variables with non-normal distribution. The Kolmogorov-Smirnov test with a 5% significance level (*P*<0.05) was used to assess the assumption of normal distribution. Student’s *t*-test, Mann-Whitney U test or chi-squared test were used to compare cohorts according to variable characteristics. Statistical analysis was performed using STATA (Stata Statistical Software: Release 16. College Station, TX: StataCorp LLC).

## RESULTS

Sociodemographic and clinical characteristics are shown in Table 1. A total of 98 patients were included, of whom 50% (n=49) underwent preoperative biliary drainage. Of this cohort, 6.1% were percutaneous or transparietohepatic and 93.9% were endoscopic. Plastic stents were the most commonly used (80.4%), followed by bare metal stents (13%) and fully covered stents (6.5%). Most patients were male (53.1%) with a mean age of 62±10 years, 28% had a history of smoking, and the median Charlson index was four in the group with preoperative biliary drainage and three in the group without drainage. 

**TABLE 1:** Socio-demographic and clinical characteristics of patients undergoing pancreaticoduodenectomy (Whipple’s surgery) according to the presence of pre-surgical biliary drainage.

	Total	Without pre-surgical biliary drainage	With pre-surgical biliary drainage	***P*-value**
Variable	n=98	n=49	n=49	
Sociodemographic characteristics				
Age, years, median (IQR)	62 (53-72)	59 (51-71)	67 (55-72)	0.11
Masculine, n (%)	52 (53.1)	19 (38.8)	33 (67.3)	0.04
BMI, median (IQR)	24 (21-26)	24 (21-26)	24 (21-26)	0.66
Smoking history, n (%)	28 (28.6)	13 (26.5)	15 (30.6)	0.74
Comorbidities, n (%)				
Arterial hypertension	43 (43.9)	22 (44.9)	21 (42.9)	1
Diabetes	24 (24.5)	10 (20.4)	14 (28.6)	0.48
History of chronic pancreatitis	6 (6.1)	4 (8.2)	2 (4.1)	0.68
History of cancer diagnosis, n (%)	7 (7.1)	4 (8.2)	3 (6.1)	1
Charlson index, median (IQR)	4 (2-4)	3 (2-4)	4 (3-5)	0.26
Functional status				
ECOG, n (%)				
0	59 (60.2)	34 (69.4)	25 (51.0)	0.38
1	37 (37.8)	15 (30.6)	22 (44.9)	
2	2 (2.0)	0 (0)	2 (4.1)	
ASA, n (%)				
1	24 (24.5)	16 (32.7)	8 (16.3)	0.26
2	72 (73.5)	31 (63.3)	41 (83.7)	
Subjective global assessment, n (%)				
0	20 (20.4)	16 (32.7)	4 (8.2)	0.11
1	38 (38.8)	15 (30.6)	23 (46.9)	
2	40 (40.8)	18 (36.7)	22 (44.9)	
Karnofsky index, median (IQR)	90 (80-100)	90 (90-100)	90 (80-100)	0.22

IQR: interquiartilic range; BMI: body mass index; ECOG: Eastern Cooperative Oncology Group classification; ASA: American Society of Anesth’esiologists classification.

Regarding functionality, the median Karnofsky index was 90 and was similar between groups. There was a higher proportion of ECOG one and two in patients with preoperative biliary drainage, but this was not statistically significant (*P*=0.38). Similarly, there were more patients with ASA >1 and subjective global assessment ≥1 in this group without reaching statistically significant differences.

Peri-procedural and procedural characteristics are shown in Table 2. Fifty-one percent of the patients had a preoperative histological diagnosis and after surgery the most common histology was adenocarcinoma, accounting for 72% of the cases. About the lesions, 45.9% were located periampullary (located ≤2 cm from greater duodenal papilla)^22^ and 43% in the pancreas, with only 6% and 4% in the common bile duct and duodenum respectively. Median preoperative bilirubin levels were higher in the preoperative biliary drainage group (median 6.4 vs 4.9 mg/ dL; *P*=0.02), as were pathological stages II and III disease (*P*=0.04). The median time from endoscopic drainage to surgery was 30 days.

**TABLE 2:** Peri-procedural and procedural characteristics.

Variable	Total	Without pre-surgical biliary drainage	With pre-surgical biliary drainage	***P-*value**
Bilirubins at admission (mg/dL). median (IQR)	5.3 (1.7-9.9)	4.9 (0.8-8.9)	6.4 (2.7-10.4)	0.02
Preoperative diagnosis. n (%)	51 (52.0)	23 (46.9)	28 (57.1)	0.69
Location of injury. n (%)				
Ampular - Periampullary	45 (45.9)	20 (40.8)	25 (51)	0.63
Head of pancreas	43 (43.9)	26 (53.1)	17 (34.7)	
Distal common bile duct	6 (6.1)	1 (2.0)	5 (10.2)	
Duodenum	4 (4.1)	2 (4.1)	2 (4.1)	
Type of shunt. n (%)				
Endoscopic	46 (46.9)	NA	46 (93.9)	NA
Percutaneous	3 (3.1)	NA	3 (6.1)	NA
Type of prosthesis in endoscopic shunt. n (%)				
Plastic	37 (37.8)	NA	37 (80.4)	NA
Fully bare metal	6 (6.1)	NA	6 (13)	NA
Fully encased metal	3 (3.1)	NA	3 (6.5)	NA
Histopathological diagnosis. n (%)				
Adenocarcinoma	72 (73.5)	30 (61.2)	42 (85.7)	0.11
Serous cystadenoma	3 (3.1)	3 (6.1)	0 (0)	
Pseudopapillary tumour	6 (6.1)	5 (10.2)	1 (2.0)	
Neuroendocrine tumour	4 (4.1)	4 (8.2)	0 (0)	
Cholangiocarcinoma	4 (4.1)	1 (2.0)	3 (6.1)	
Myoepithelial Hamartoma	2 (2.0)	1 (2.0)	1 (2.0)	
Intraductal Mucinous Papillary Intraductal Neoplasm	2 (2.0)	2 (4.1)	0 (0)	
Chronic pancreatitis	4 (4.1)	2 (4.1)	2 (4.1)	
Tubular adenoma with high grade dysplasia	1 (1.0)	1 (2.0)	0 (0)	
Lymphadenectomy. n (%)	97	48 (98)	49 (100)	1
Stage. n (%)				
Not applicable	17 (17.3)	14 (28.6)	3 (6.1)	0.04
I	19 (19.4)	11 (22.4)	8 (16.3)	
II	37 (37.8)	17 (34.7)	20 (40.8)	
III	25 (25.5)	7 (14.3)	18 (36.7)	
Time from endoscopic drainage to surgical management. Median (IQR)	30 (14.5-69.5)	NA	30 (14.5-69.5)	NA

IQR: interquiartilic range.

Operative and postoperative outcomes are shown in Table 3. There were no differences in intraoperative transfusion requirements (*P*=0.64), intraoperative complications (*P*=0.34) or postoperative complications according to the Clavien-Dindo classification (*P*=0.15). Similarly, no differences were found in surgical site infection, biliary leak, pancreatic fistula, multi-organ failure, need for mechanical ventilation in the post-operative period, intensive care unit stay or need for unplanned surgical reintervention. 

**TABLE 3:** Complications in patients undergoing pancreatoduodenectomy (Whipple’s procedure) according to the presence of pre-surgical biliary drainage.

	Total	Without pre-surgical biliary drainage	With pre-surgical biliary drainage	***P*-value**
Variable	n=98	n=49	n=49	
Intraoperative transfusion requirement. n (%)	11 (11.2)	5 (10.2)	6 (12.2)	0.64
Intraoperative complications. n (%)	12 (12.2)	7 (14.3)	5 (10.2)	0.34
Postoperative complications. n (%)	55 (56.1)	30 (61.2)	25 (51.0)	0.15
Clavien-Dindo I	9 (9.2)	7 (14.3)	2 (4.1)	0.91
Clavien-Dindo II	16 (16.3)	8 (16.3)	8 (16.3)	
Clavien-Dindo III	14 (14.3)	8 (16.3)	6 (12.2)	
Clavien-Dindo IV	16 (16.3)	7 (14.3)	9 (18.4)	
Operative site infection. n (%)	29 (29.6)	18 (36.7)	11 (22.4)	0.18
Superficial	3 (3.1)	2 (4.1)	1 (2.0)	1
Deep	1 (1.0)	1 (2.0)	0 (0)	
Organ space	25 (25.5)	15 (30.6)	10 (20.4)	
Biliary leak. n (%)	11 (11.2)	4 (8.2)	7 (14.3)	0.12
Pancreatic fistula. n (%)	23 (23.5)	11 (22.4)	12 (24.5)	
A	5 (5.1)	2 (4.1)	3 (6.1)	1
B	7 (7.1)	5 (10.2)	2 (4.1)	
C	11 (11.2)	4 (8.2)	7 (14.3)	
Multi-organ failure. n (%)	7 (7.1)	3 (6.1)	4 (8.2)	1
Postoperative IMV requirement. n (%)	3 (3.1)	2 (4.1)	1 (2.0)	0.29
Need for ICU stay. n (%)	25 (25.5)	10 (20.4)	15 (30.6)	0.12
Unplanned re-operation requirement 30 days. n (%)	22 (22.4)	10 (20.4)	12 (24.5)	0.5
Unplanned readmission requirement 30 days. n (%)	9 (9.2)	8 (16.3)	1 (2.0)	0.02
Death 30 days postoperative. n (%)	14 (14.3)	4 (8.2)	10 (20.4)	0.03
Overall hospital stay. median (IQR)	19.5 (11-31)	23 (12-37)	16 (10-26)	0.05
Median postoperative hospital stay. median (IQR)	13 (9-23)	16 (11-39)	11 (9-18)	0.03

IQR: interquiartilic range; IMV: invasive mechanical ventilation; ICU: intensive care unit.

There was a higher 30-day mortality in the preoperative biliary drainage group (20.4% vs 8.2%; *P*=0.03). Deceased patients from each cohort were compared according to preoperatory biliary drainage. Causes of death were similar in both groups. Although no statistically differences were seen between both groups, older age, lower functional status and more frequent complications were found compared with non-decea­sed patients (SUPPLEMENTARY TABLE 1).

**SUPPLEMENTARY TABLE 1.** Socio-demographic. clinical. procedural and complications characteristics of patients undergoing pancreaticoduodenectomy (Whipple’s surgery) according to the presence of pre-surgical biliary drainage in deceased patients 30 days postoperative.

	Total	Without pre-surgical biliary drainage	With pre-surgical biliary drainage	***P*-value**
Variable	n=14	n=4	n=10	
Age. years. median (IQR)	74.5 (63-77.5)	77.5 (73.2-78.7)	71 (62.5-76)	0.24
Masculine. n (%)	9 (64.3)	2 (50)	7 (70)	0.58
Charlson index. median (IQR)	4 (2-5)	3.5 (2-5)	4 (2-4)	0.14
ECOG. n (%)				
0	5 (35.7)	1 (25)	4 (40)	1
1	9 (64.3)	3 (75)	6 (60)	
2	0 (0)	0 (0)	0 (0)	
ASA. n (%)				
1	2 (14.3)	1 (25)	1 (10)	0.73
2	12 (85.7)	3 (75)	9 (90)	
Subjective global assessment. n (%)				
0	1 (7.1)	1 (25)	0 (0)	0.94
1	3 (21.4)	0 (0)	3 (30)	
2	10 (71.4)	3 (75)	7 (70)	
Karnofsky index. median (IQR)	85 (80-90)	85 (80-97.5)	85 (80-90)	0.84
Bilirubins at admission (mg/dL). median (IQR)	7.9 (3.5-19.5)	18.8 (9-28.7)	5.9 (2.7-14.7)	0.18
Stage. n (%)				
Not applicable	1 (7.1)	0 (0)	1 (10)	0.66
I	5 (35.7)	2 (50)	3 (30)	
II	6 (42.9)	1 (25)	5 (50)	
III	2 (14.3)	1 (25)	1 (10)	
Time from endoscopic drainage to surgical management. Median (IQR)	24.5 (12.5-109.5)	NA	24.5 (12.5-109.5)	NA
Intraoperative complications. n (%)	5 (35.7)	2 (50)	3 (30)	0.58
Postoperative complications. n (%)	8 (57.1)	1 (25)	7 (70)	0.24
Clavien-Dindo IV	8 (57.1)	1 (25)	7 (70)	0.24
Operative site infection. n (%)	5 (35.7)	1 (25)	4 (40)	1
Organ space	5 (35.7)	1(25)	4 (40)	1
Pancreatic fistula. n (%)	5 (35.7)	1 (25)	4 (40)	1
A	1 (7.1)	0 (0)	1 (10)	1
C	4 (28.6)	3 (75)	3 (30)	
Multi-organ failure. n (%)	6 (42.9)	2 (50)	4 (40)	1
Need for ICU stay. n (%)	11 (78.6)	3 (75)	8 (80)	1
Unplanned re-operation requirement 30 days. n (%)	7 (50)	2 (50)	5 (50)	1
Unplanned readmission requirement 30 days. n (%)	1 (7.1)	1 (25)	0 (0)	0.29
Overall hospital stay. median (IQR)	13.5 (8-25)	16.5 (10-23)	13.5 (5-25)	0.73
Median postoperative hospital stay. median (IQR)	7 (2-11)	0.5 (0-1)	7.5 (3-11)	0.54
Cause of death. n (%)				
Sepsis	10 (71.4)	3 (75)	7 (70)	0.85
Cardiovascular complications	2 (14.3)	0 (0)	2 (20)	0.84
Intraoperative death	1 (7)	1 (25)	0 (0)	0.42
Other	1 (7)	0 (0)	1 (10)	0.59

IQR: interquartilic range; ECOG: Eastern Cooperative Oncology Group classification; ASA: American Society of Anesthesiologists classification; NA: not applicable; ICU: i’ntensive care unit.

The non-drainage group had a longer postoperative hospital stay (16 vs 11 days; *P*=0.03) and a higher need for hospital readmission at 30 days (*P*=0.02). This last finding represents a total of eighth patients, of which 50% (n=4) were for residual abdominal collections treated with oral antibiotics and 25% (n=2) for type A pancreatic fistulas. None of these readmissions had a hospital stay longer than 96 hours. The remaining 25% (n=2) of readmissions were for complications resulting in death.

## DISCUSSION

Whipple procedure is a therapeutic tool with curative potential in periampullary tumours and pancreatic cancer. The latter is associated with a high mortality rate and a low survival rate of approximately 9% at 5 years^23^ ^,^ ^24^, being the seventh leading cause of cancer death in developed countries and the sixth most lethal tumors in Colombia, according to GLOBOCAN data from 2020^25^. Given the significant morbidity associated with the procedure, strategies such as preoperative biliary drainage have been developed to improve outcomes, with the result that in current clinical practice up to 50% of patients referred to tertiary centers undergo preoperative biliary drainage, and as many as 75% of them before being evaluated for surgery^26^ ^,^ ^27^.

In this study, we retrospectively evaluated outcomes according to the presence of preoperative biliary drainage in patients undergoing Whipple’s procedure regardless of the cause. Analysis of our data shows that 50% of patients undergo preoperative biliary drainage. This cohort had higher bilirubin levels on admission, probably related to a higher proportion of patients with stage II-III pathology, which could be a determinant in the decision to take patients directly to surgery or at a later stage after biliary drainage. Although there was a trend towards a greater burden of comorbidities as measured by objective scales and worse ECOG scores in the biliary drainage group, this did not reach statistical significance. 

The percentage of patients referred for preoperative biliary drainage and the differences in baseline clinical characteristics in our results are similar to those previously reported in the literature and reveal some selection bias, as patients referred for preoperative biliary drainage also have more advanced disease stages, a pathological stage that is the main determinant of survival in resectable tumours^28^ ^-^ ^32^. 

Despite the pathophysiological phenomena and deleterious effects of jaundice on cellular immunity, nutritional status and the balance between procoagulant and anticoagulant factors, which have been shown to be reversible after preoperative biliary drainage even in animal models^33^ ^,^ ^34^, this does not correlate conclusively with clinical outcomes. Some studies have left open the discussion whether jaundice itself is a factor directly related to outcomes; Macias et al., described in a retrospective study of 71 patients that preoperative biliary drainage was a factor that negatively impacted survival (Hazard ratio, 0.51; *P*=0.016), however, of the total cohort 80% (n=62) were jaundiced and 53% were taken for preoperative biliary drainage without clearly discriminating what proportion of patients taken directly to surgery had obstructive jaundice^29^. Van Der Gaag et al., in the DROP trial study, included only patients with total bilirubin levels between 2.3 and 14.6 mg/dL^8^, leaving open the question of whether the results are applicable to populations with higher bilirubin levels in whom there is greater theoretical support for performing biliary drainage as some studies have shown^12^.

Our study showed that there were no significant differences in intraoperative and postoperative complications, including intraoperative transfusion requirement, biliary leak, pancreatic fistula, multiorgan failure, need for postoperative mechanical ventilation, intensive care unit stay, or need for unplanned surgical reintervention. However, a higher number of unplanned hospital readmissions were observed in the cohort of patients without preoperative biliary drainage, mainly due to complications requiring no more than 96 hours of in-hospital care. The same group also had a longer hospital stay. This is probably because after biliary drainage, most patients were discharged for surgery with a planned readmission, which contributed to the shorter length of stay. These results show no benefit in terms of postoperative complications, in line with most of the current literature where even preoperative biliary drainage has been shown to increase the risk of complications.

Although there were no differences in postoperative complications and ISO between groups in our study, similar to the reported in other trials and systematic reviews^12^ ^,^ ^35^, different outcomes have been described by other authors. Gavazzi et al. described a higher incidence of deep ISO in patients with preoperative biliary drainage (*P*=0.044)^36^; a similar result that the observed in a systematic review and meta-analysis of 36 studies in which there were higher rates of surgical wound infection (*P*<0.005) and positive biliary culture (*P*<0.0001), the latter increasing postoperative mortality and morbidity^37^. On the other hand, the DROP trial documented a higher rate of complications in the biliary bypass group (74% vs 39%; *P*=<0.001), complications mainly related to biliary drainage (e.g. pancreatitis, cholangitis, perforation) and not to surgery (47% vs 37%; RR 0.79; 95%CI 0.57 to 1.11; *P*=0.14)^8^. A meta-analysis published in 2017 that included 25 studies showed an increased incidence of complications (OR: 1.40; 95%CI: 1.14-1.72; *P*=0.002) and ISO (OR: 1.94; 95%CI: 1.48-2.53; *P*<0.00001) compared to patients taken directly to surgery^38^. 

Our data show that there was higher mortality in the pre-surgical biliary bypass group (10% vs 4%; *P*=0.03). Some studies show a tendency to increase mortality in patients undergoing biliary drainage, however, without statistical significance^8^ ^,^ ^35^ ^,^ ^38^. In our study, there were no significant differences between baseline characteristics, tumor staging or complications when comparing deceased patients in both groups. However, older age, lower functional status and more complicated were evident compared with non-deceased patients (SUPPLEMENTARY TABLE 1). However, small sample size could explain no significant differences. However, we hypothesize mortality difference could be explained by several factors, including: (1) a higher proportion of neoplasms with more advanced pathological stages in this group of patients; (2) the time between drainage and surgery, since it has been described that a time interval >21 days is an independent prognostic factor in survival^39^. In our study it was 30 days, probably related to barriers to healthcare access; (3) A possible selection bias, as there is tendency for worse comorbidities and functionality in patients with drainage compared to those who are taken directly to surgery. On the other hand, the main cause of death was sepsis, followed by cardiovascular complications. There were no differences between the two groups. However, the causes of death are multifactorial. Nutritional and functional status could be confounding or interacting variables for death. Selection of appropriate patients for the Whipple procedure is therefore suggested.

This is the first Latin American study describing the experience in the treatment of resectable periampullary and pancreatic tumors, contributing to the literature new data on the outcomes associated with preoperative biliary drainage. Our results suggest, as do retrospective and prospective studies to date, that there is no benefit to preoperative biliary drainage. However, there are limitations that we must acknowledge. Our study is descriptive and retrospective, and therefore does not allow conclusions of causality, and is limited to presenting differences in outcomes between groups. Multicenter, prospective, randomized, prospective studies with a larger sample size will be required to define the true impact of preoperative biliary drainage. Additionally, within the analysis there was a small proportion of patients with percutaneous biliary drainage (three patients) and with metallic biliary prostheses (nine patients), so the validity of our results in populations with this type of devices should be studied in future studies. Finally, cohorts’ comparison suggests differences between patient’s clinical characteristics. Preoperatory drainage cohort presents lower functional status (although not statistically significant) which could determine the decision of not performing surgery and, therefore, performing biliary drainage instead. Clinical studies in which surgical decision is addressed are recommended. 

## CONCLUSION

This study suggests that preoperative biliary drainage in resectable periampullary and pancreatic tumors does not affect the rate of intraoperative and postoperative complications. The differences found, with higher mortality rate in patients with preoperative biliary drainage versus patients who are taken directly to surgery may be associated with differences in the baseline characteristics of the patients in each group and/or delays between drainage and surgery. Further studies with prospective design are needed in the future to clarify the effects of presurgical biliary drainage in these patients. According to authors opinion, preoperatory biliary drainage should be decided on a multidisciplinary basis according to local resources, cancer staging, patient functional status and surgical expertise on Whipple procedure.
